# Consumption Localities

**Published:** 1857-08

**Authors:** 


### CONSUMPTION LOCALITIES.

From information derived from many portions of the United States, the conclusion is clearly warranted, that all low, damp places facilitate the development of consumption of the lungs.

On the other hand, high and airy situations are largely exempted from this malady. The city of Mexico is many thousand feet above the level of the sea, and th[illegible] deaths there, from consumption, than in any city [illegible] on the globe, of which we are informed.

The practical conclusion therefore is, that hilly countries, being high and dry, should be sought out by the consumptive. If this is a legitimate conclusion, then the last place in the world for a man to go, who is laboring under consumption, is Florida, Louisiana, or any other flat, wet country, north or south.—*See Hand-Book of Consumption.*

